# Vinylimidazole-Based Polymer Electrolytes with Superior Conductivity and Promising Electrochemical Performance for Calcium Batteries

**DOI:** 10.1021/acsapm.2c01140

**Published:** 2022-09-12

**Authors:** Shreyas Pathreeker, Ian D. Hosein

**Affiliations:** Department of Biomedical and Chemical Engineering, Syracuse University, Syracuse, New York 13244, United States

**Keywords:** calcium, electrolytes, polymers, salts, energy, batteries

## Abstract

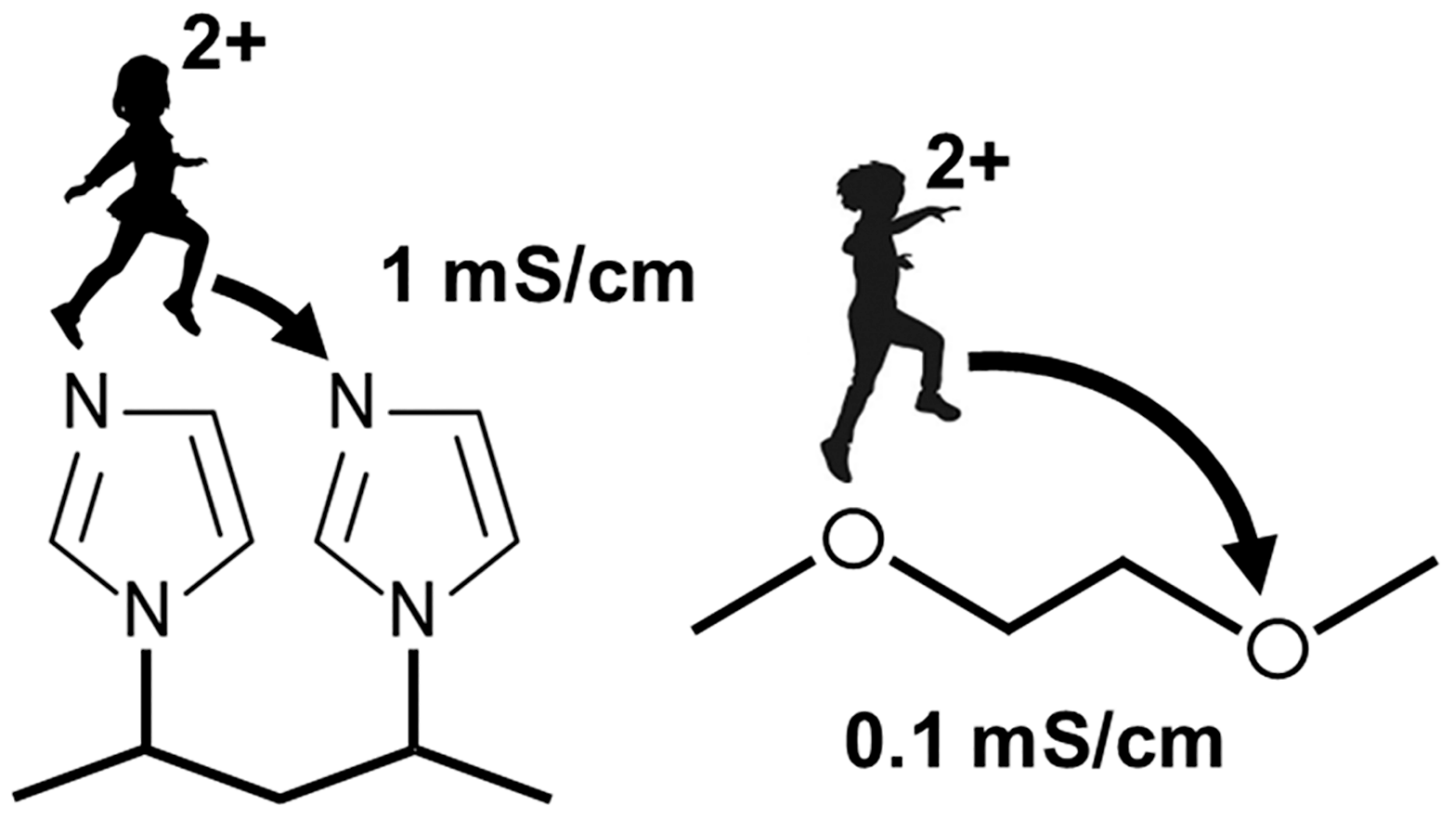

Calcium batteries are next-generation energy storage technologies with promising techno-economic benefits. However, performance bottlenecks associated with conventional electrolytes with oxygen-based coordination chemistries must be overcome to enable faster cation transport. Here, we report an imidazole-based polymer electrolyte with the highest reported conductivity and promising electrochemical properties. The polymerization of vinylimidazole in the presence of calcium bis(trifluoromethanesulfonyl)imide (Ca(TFSI)_2_) salt creates a gel electrolyte comprising a polyvinyl imidazole (PVIm) host infused with vinylimidazole liquid. Calcium ions effectively coordinate with imidazole groups, and the electrolytes present room temperature conductivities of >1 mS/cm. Reversible redox activity in symmetric Ca cells is demonstrated at 2 V overpotentials, stable cycles at 0.1 mA/cm^2^, and areal capacities of 0.1 mAh/cm^2^. Softer coordination, polarizability, and closer coordinating site distances of the imidazole groups can explain the enhanced properties. Hence, imidazole is a suitable chemical benchmark for the future design and advancement of polymer electrolytes for calcium batteries.

Calcium batteries are emerging as a promising next-generation energy storage technology. They will offer numerous benefits including the use of calcium metal with significantly greater mineral abundance, opportunities for domestic supply (mitigating global supply chain concerns), and favorable economics associated with lower cost as well as energy density and rate capabilities comparable to state-of-the-art lithium batteries.^1^ To keep pace with current advances in lithium metal battery technology, the development of high performance polymer electrolytes is critical to the realization of practical and desired solid or quasi-solid state calcium metal batteries, especially to support safe battery operation in high demand applications, such as electric vehicles and even grid scale storage.

Electrolyte chemistries for calcium-ion conduction have focused primarily on oxygen-based coordination chemistry, owing to their proven suitability for other systems, primarily due to the highly polar ether O atoms that enable good salt dissociation.^2,3^ This has inspired analogous calcium electrolytes, which use common carbonate-^4−6^ or ether-based^7^ solvents and predominately ether-based polymer backbones,^8−10^ with ionic moieties being one exception.^11−13^ In the case of polymer electrolytes, however, ether-containing backbones result in low ionic conductivity and difficulties with achieving high redox rates in electrochemical cells.^14^ This is fundamentally associated, in part, to the calcium ion’s combined large ionic radius and greater charge density resulting in strong coordination to oxygen, as compared to other cations,^15,16^ in addition to the large hopping distance between neighboring coordinating sites for a poly(ethylene oxide) (PEO) chain.^17^ The incorporation of polarizable units in the polymer backbone and softening cation–polymer interactions are some of the potential strategies to overcome challenges encountered with ether-based multivalent polymer electrolytes.^18^ Hence, to advance the coordination properties of calcium ions, more suitable coordinating chemistries are required that yield greater conductivity and more facile electrochemical kinetics.

In this work, we present an imidazole–pendant polymer host (see Scheme 1) with superior electrolyte properties for calcium ion conduction. Bis(trifluoromethanesulfonyl)imide (Ca(TFSI)_2_) is used as the salt due to the highly delocalized TFSI^–^ anion, which facilitates good salt dissociation.^5^ A high dielectric constant and polarity of the solvent are also important for improving salt dissociation in battery electrolytes. A primary reason behind the popularity of PEO is the highly polar ether O atom. Table 1 provides comparative values between the ether and the imidazole moieties in terms of dielectric constant, donor number, and dipole moment. The imidazole group is a planar 5-membered ring consisting of two nitrogen atoms and shows the capability of coordinating well to cations,^21−26^ owing to the high donor capability among its class of N-containing 5 and 6 member rings (e.g., similar structures such as 3-methylpyridine and 4-methylpyridine possess donor numbers of 36 and 39, respectively). The imidazole moiety also presents a more delocalized electron donating group (i.e., polarizable), which can offset the strong complexation properties of Ca^2+^. Thereby, it can reduce solvation strengths of the coordination environments (i.e., provide softer imidazole–cation coordination), while still enabling salt dissociation and lower energetic barriers for cation transport via facile hopping among coordinating sites. Additionally, via the free-radical polymerization of a vinylimidazole monomer, we furthermore tether the imidazole groups as pendants on a backbone, thereby constructing a coordination environment with the pendant imidazole coordinating sites held near one another, facilitating more facile transport of calcium ions. This combination of softer coordination and closer coordinating sites can result in a surprising increase in the ionic conductivity over pure imidazole electrolyte as well as easily achievable redox activity in Ca//Ca electrochemical cells at promising current densities. This is the first instance of the study of imidazole-based polymer electrolytes for calcium ion conduction, whose attractive conductive and electrochemical properties signify a paradigm shift in the predominate chemistry space for the polymer electrolyte design of calcium batteries.

**Table 1:** Dielectric Constants, Donor Numbers, and Dipole Moments for the Ether Moiety and the Imidazole Moiety

moiety	dielectric constant	donor number	dipole moment (D)
ether	5.0^2^	19.2^a^^,^^19^	1.15
imidazole	12.0^20^	36^b^^,^^21^	3.67

a Value taken from diethyl ether.

b Value taken from PVIm in pyridine.

We first investigated the effect of salt incorporation into the vinylimidazole monomer liquid. Figure 1 shows deconvoluted FTIR spectra for liquid formulations (i.e., vinyimidazole monomer + salt) and their corresponding polymers obtained after photopolymerization. Hereon, polymerized samples are denoted as PVIm–0.1, PVIm–0.5, and PVIm–1.0, wherein the numbers denote the molar concentration of salt used. Full FTIR spectra of the pure monomer, pure salt, liquid mixtures before polymerization, and samples after polymerization are provided in Figure S1. The mode associated with the combined imidazole ring C=N bond stretching and C–H (ring) bond^21,23^ bending centered at ∼1225 cm^–1^ is chosen for coordination analysis. Upon addition of salt, two features are detected for all liquid resins and all polymer samples: (1) the primary imidazole peak at ∼1225 cm^–1^ and (2) a higher–wavenumber shoulder at ∼1231 cm^–1^. We first focus on the coordinated monomers (first column). Since this shoulder was not detected in the neat monomer, its presence upon the addition of salt indicates the formation of a new coordination environment for the imidazole. To detect possible overlap of this shoulder with the salt anion band, the FTIR spectrum of the salt was used as a baseline, wherein a broad peak at ∼1225 cm^–1^ was observed (Figure S2a). However, this peak did not overlap with the shoulder formed at 1231 cm^–1^. Additional analysis based on the 960 cm^–1^ peak attributed to the imidazole ring also revealed the formation of shoulder peaks upon the addition of salt (Figure S2b), similar to the 1231 cm^–1^ peak, which indicates that these shoulders originate from imidazole–Ca^2+^ coordination. Additionally, a new peak was detected at ∼926 cm^–1^ upon addition of salt to the monomer (Figure S2c), which has been reported previously in the literature for a system consisting of 1-vinylimidazole and CaTf_2_N salt.^25^ The coordination environment is explained as follows: the pyridine-type N atom of the imidazole ring possesses one lone pair of electrons and thus behaves as a ligand toward bivalent metal ions such as Zn^2+^ and Ni^2+^ to form stable metal–ligand complexes.^21,24^ As such, the addition of Ca(TFSI)_2_ salt to the vinylimidazole monomer is expected to form N–Ca^2+^ coordination complexes, wherein the N atoms behave as Lewis bases (electron donors) and the Ca^2+^ ions, as Lewis acids (electron acceptors). Therefore, the shoulder corresponds to a population of imidazole groups involved in this coordination environment and confirms that the addition of salt results in successful monomer (imidazole)–Ca^2+^ coordination. This is important since monomer–Ca^2+^ coordination implies salt dissociation, which is necessary for cation transport in polymer electrolytes. Relative to the neat monomer (∼1224 cm^–1^), mild shifts in the free monomer (i.e., uncoordinated imidazole groups) peak in the resins are also detected for all salt concentrations, which also indicates N–Ca^2+^ coordination. The shoulder becomes more prominent with an increase in salt concentration, indicating a higher degree of N–Ca^2+^ coordination; i.e., more N atoms from the imidazole ring coordinate with the Ca^2+^ ions from the salt owing to a higher number of the latter.

**Figure 1:**
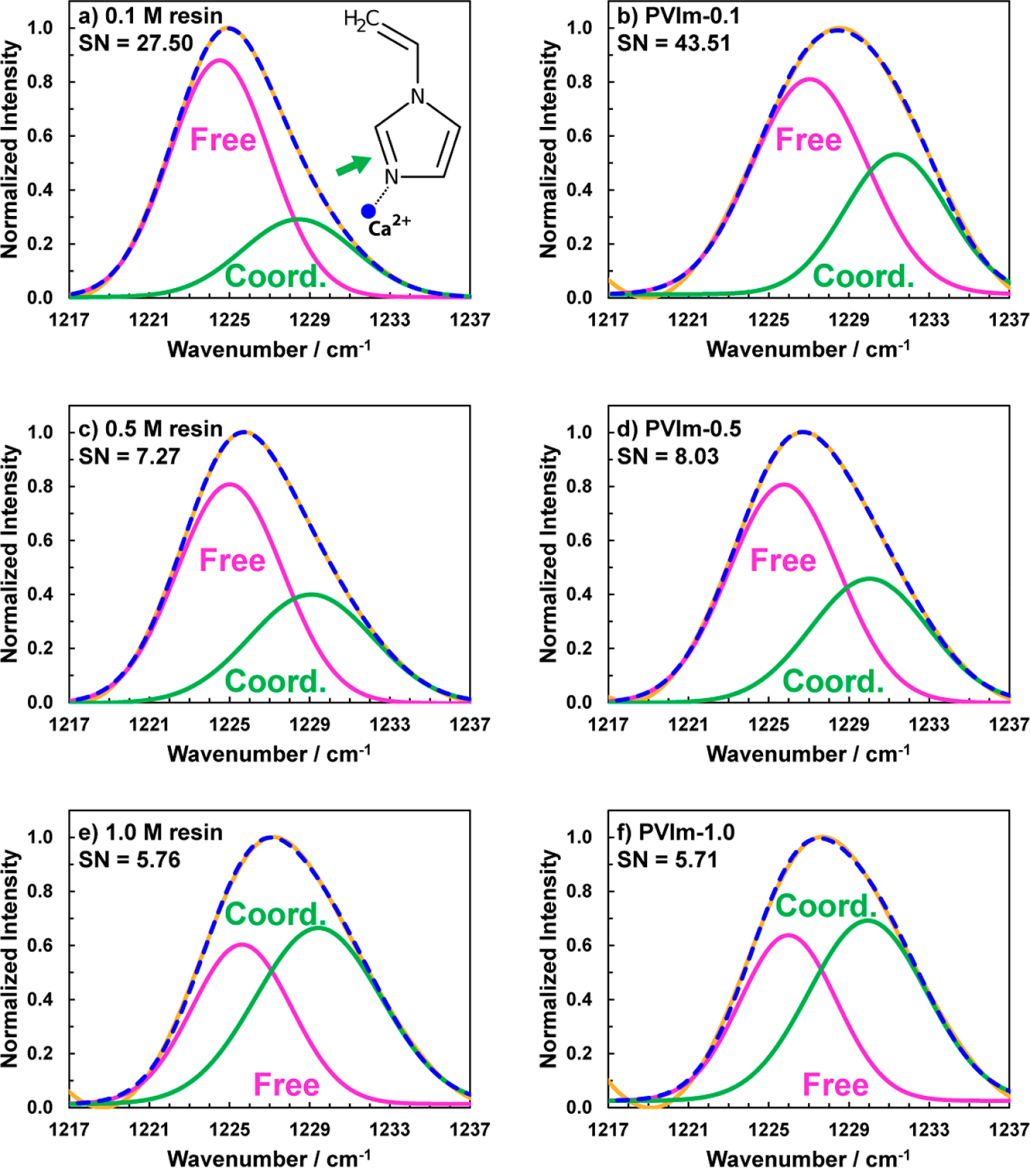
Deconvoluted FTIR spectra representing the C=N stretching bond of the imidazole ring seen at ∼1225 cm^–1^. Data is shown for (a, c, e) liquid mixtures prior to photopolymerization and (b, d, f) polymers formulated with different Ca(TFSI)_2_ salt concentrations. The dashed blue line is the sum of all fits, and the solid yellow line is the experimental data. The large green peaks indicate more coordination of calcium ions with imidazole with an increase in salt concentration. Solvation numbers for the liquid formulations (resins) are also indicated.

Upon photopolymerization (second column in Figure 1), the same shoulder (∼1231 cm^–1^) as in the coordinated monomers is observed for all salt concentrations, yet their intensities are found to be higher than in their respective liquid resins, which indicates that polymerization results in higher imidazole–salt coordination. Solvation numbers (SNs) were calculated for the resins and the polymers. From liquid to polymer, the SN increased for 0.1 and 0.5 M salt concentrations but decreased slightly for the 1.0 M salt concentration. The SNs of 6–8 observed here are in good agreement with previous results,^5,16^ whereas the higher SN for the 0.1 M salt concentration is an artifact of the dilute salt concentration and an overestimation of solvation by FTIR. Apparent coordination numbers of a similar magnitude have been reported in the literature.^27^ The free imidazole peak is also found to shift (Figure 1, PVIm–0.1 vs PVIm–0.5 M and PVIm–1.0), further suggesting a higher degree of N–Ca^2+^ coordination upon photopolymerization compared to their liquid counterparts (not for PVIm–1.0). This shift may be due to the constraints placed on the pendant groups due to polymerization-induced shrinkage. Two plausible explanations can be provided for the increase in imidazole–Ca^2+^ coordination going from liquid to polymer: (1) the ability of triflimide salt cations to form physical cross-links^25^ more easily between imidazole pendant groups now “tethered” along the polymer chains as opposed to the free form imidazole groups in the liquid state as well as (2) the associated densification upon polymerization, which also brings pendant groups closer together. As more salt is added to the polymer, the percentage of unpaired TFSI^–^ anions remain at ∼50%, but imidazole–Ca^2+^ coordination increases, implying that excess Ca^2+^ must coordinate with the imidazole. Coordination of Ca^2+^ with TFSI^–^ is also revealed by deconvolution analysis presented for the salt (see Figure S3).

Figure 2 provides quantitative analysis based on the FTIR data for the degree of coordination for the neat monomer and polymerized systems. For comparison, the percentage of free (uncoordinated TFSI^–^) anions is also provided. Two key aspects of the coordination of these imidazole-based monomer/polymer electrolytes are evident: (1) a strong percentage of N–Ca^2+^ coordination for all salt concentrations with an approximate increase with an increase in salt concentration and (2) the consistent free TFSI^–^, which suggests that, as the concentration of salt increases, the percentage of free TFSI^–^ is always 50% and Ca^2+^ must necessarily coordinate with more imidazole in the polymer with an increase in salt concentration. To better understand the role played by the Ca(TFSI)_2_ salt concentration on imidazole–Ca^2+^ coordination, the out-of-plane vibration mode associated with the O=S=O bond centered at 1136 cm^–1^ for free TFSI^–^ anions^28^ was analyzed. It is found that, overall, ∼55% of the TFSI^–^ anions are free (i.e., unpaired), while ∼45% of the TFSI^–^ anions are paired with Ca^2+^ ions. To explain this partial dissociation of the salt molecule, we propose a monodissociated TFSI^*–*^ anion in the form CaTFSI^*+*^ and TFSI^*–*^. A plausible explanation for this type of dissociation could be the relatively bulky nature of the imidazole pendant groups, which are unable to penetrate the ionic volume between Ca^*2+*^*and TFSI*^*–*^ and fully separate the two. Upon polymerization, the percentage of free TFSI^–^ anions increases slightly for 0.1 and 0.5 M salt concentrations, indicating that more Ca^2+^ has dissociated from the salt upon polymerization. We note that, for accurate quantitative estimation of charge carriers in the electrolyte, the percentages shown here must be analyzed in conjunction with the numerical values of the salt concentrations. On a molar basis, 0.11 M TFSI^–^ (55% of 0.1 M, 2 TFSI^–^ due to Ca(TFSI)_2_) is free in PVIm–0.1 and, accordingly, 0.56 M TFSI^–^, in PVIm–0.5 and 1.12 M TFSI^–^, in PVIm–1.0. These free TFSI^–^ numbers are indirect indicators of free Ca^2+^ freely available for coordination to the polymer: on a basis of 1 Ca^2+^ cation for every 2 TFSI^–^ anions, there is 0.01 M free Ca^2+^ in PVIm–0.1, 0.065 M free Ca^2+^ in PVIm–0.5, and 0.12 M free Ca^2+^ in PVIm–1.0. The Ca^2+^ ions are expected to coordinate with the pyridine N atom of the imidazole ring, since the free TFSI^–^ is unlikely to coordinate with the polymer owing to its bulky nature and the associated steric effects.^29^ Therefore, an increase in salt concentration leads to an increase in imidazole–Ca^2+^ coordination owing to an increase in the number of free Ca^2+^ ions in the electrolytes. The apparent low concentration of free Ca^2+^ ions may be due to the choice of the peaks used for coordination analysis, as well as the partially dissociated salt as proposed above. Regarding the former, the 740 cm^–1^ peak belonging to the S–N–S vibrational mode is more suitable for coordination analysis but could not be used in this work owing to its overlap with the monomer (see Supporting Information). However, the anion peaks used herein are still reliable and effective for the purpose of coordination analysis.^30,31^

**Figure 2:**
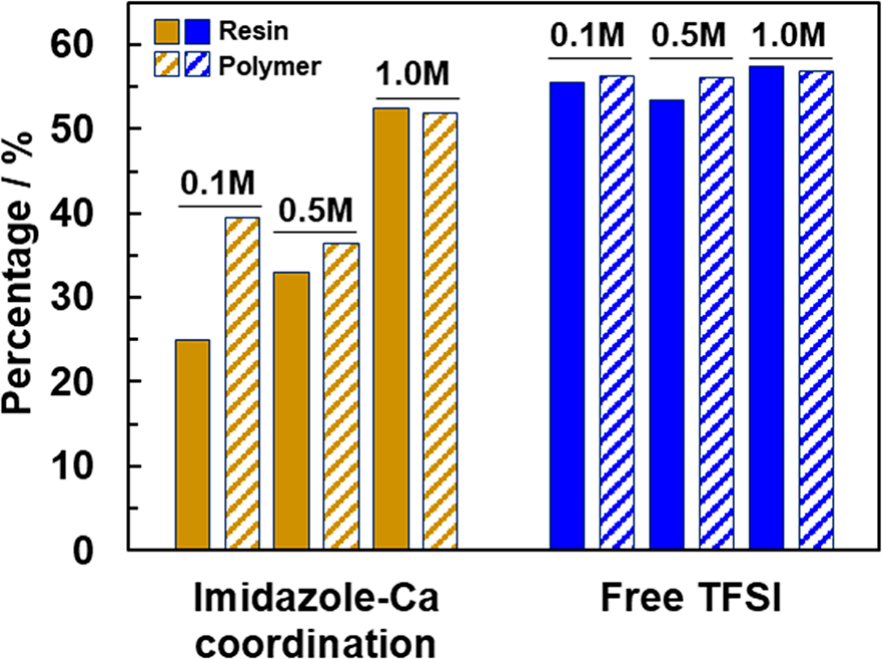
Percent coordination calculated from deconvoluted FTIR peak intensities. The 1231 cm^–1^ peak (C–N stretching) was used for the 1-vinylimidazole, whereas the peak centered near 1132 cm^–1^ was used for the free TFSI (SO_2_).

Thermogravimetric analysis of the polymer electrolytes revealed mass loss beginning immediately after 70 °C and ending at ∼260 °C for both salt concentrations. 58% of the initial mass was lost for PVIm–0.5, whereas 44% of the initial mass was lost for the PVIm–1.0. These values are associated with the loss of unreacted vinylimidazole monomer from the polymer electrolytes (see Figure S4). Hence, the polymer electrolytes are structurally gel-like material (i.e., gel polymer electrolyte, GPE) composed of liquid 1-vinylimidazole solvent (with the R group being C=C) in the presence of the polyvinyl imidazole (PVIm) host. The smaller amount of unreacted monomer in PVIm–1.0 is expected due to the higher degree of cross-linking caused by the increased salt concentration as confirmed by FTIR analysis, which also increases the overall monomer conversion. The sharp loss in mass detected at 380 °C for both salt concentrations corresponds to the thermal degradation of PVIm polymer via depolymerization and chain scission.^32^ In the temperature range explored, the analysis of polymer structure via differential scanning calorimetry (DSC) revealed no discernible glass transition temperature for either salt concentration (2nd heating), indicating that the polymer is in a glassy state at room temperature (see Figure S5). This is expected due to the cross-linking of the pendant imidazole groups by Ca^2+^ as mentioned previously. For reference, the *T*_g_ for neat polymer (i.e., no salt, PVIm–0.0) was ∼165 °C, consistent with previous studies.^32^ X-ray diffraction (XRD) analysis of the neat monomer and as-synthesized polymers between 10° and 80° revealed no crystalline peaks, indicating the amorphous nature of the polymers and complete dissolution of the salt (see Figure S6).

Molecular weights of PVIm–0.5 and PVIm–1.0 were determined using gel permeation chromatography (GPC), and are listed in Table 2. Molecular weights of PVIm–1.0 were between 2.5 and 4 times higher than those for PVIm–0.5 due to the cross-linking effect of salt and lower monomer content as evidenced by the FTIR and thermogravimetric analysis (TGA) analyses, respectively. Overall, the obtained polymer electrolytes comprise a liquid phase and a solid polymer phase, wherein the pendant imidazole groups are coordinated with Ca^2+^ from the salt, thereby offering a suitable environment for calcium ion conduction. Subsequent polymerization of the remnant monomer is possible but highly unlikely in this system because of two key reasons: (1) the short lifetime of radicals and (2) the inability of Irgacure 784 to initiate thermal polymerization to the best of our knowledge.

**Table 2:** GPC-Derived Molecular Weights of PVIm–0.5 and PVIm–1.0

sample	*M*_n_ (kg/mol)	*M*_w_ (kg/mol)	*M*_v_ (kg/mol)	PDI
PVIm–0.5	10.17	13.32	16.21	1.31
PVIm–1.0	26.03	40.59	65.85	1.56

Electrochemical characterization of these polymer electrolytes is shown in Figure 3. Nyquist plots are shown in Figure 3a for the lower bound and upper bound temperatures of the conductivity measurements (25 and 70 °C, respectively) for both salt concentrations. The room temperature impedance for PVIm–1.0 (680 Ω) is higher than that for PVIm–0.5 (390 Ω). The cationic transference number (PVIm–0.5) was measured to be ∼0.31. For comparison, room temperature impedance of the neat polymer (without salt) was 36 000 Ω, giving an ionic conductivity of 1.7 × 10^–5^ S/cm (see Figure S7). Upon an increase of the temperature to 70 °C, the semicircles disappear and the impedances associated with both polymer electrolytes decrease significantly to ∼180 Ω. The total cell impedances at room temperature are high owing to interfacial effects at the electrolyte–electrode interface. For both salt concentrations, these cell impedances decrease considerably with an increase in temperature owing to softening of the polymer at the electrode–polymer interface and improved interfacial contact. The Arrhenius-like behavior of the conductivity vs 1000/*T* plot is evident for both salt concentrations, as shown in Figure 3b, and suggests that ion conduction in the polymer electrolytes occurs primarily via the ion hopping mechanism. This is associated with transport of cations in the liquid electrolyte domains within the polymer matrix as well as intrachain hopping along the polymer chain pendant groups. Therefore, it is possible that, besides hopping between tethered imidazole pendant groups, cation motion can also occur via vehicular transport. Due to the coexistence of liquid domains and glassy polymer in the electrolytes, the Arrhenius mechanism would still hold for this system, which is reflected in the conductivity data. The influence of segmental motion of the polymer can be neglected owing to its glassy nature as revealed by DSC analysis. Some ambiguity of the ionic conductivity between 25 and 30 °C is observed for PVIm−1.0, which may be attributed to the initial non-uniform heating of the sample. Overall, the high ionic conductivity of the entrapped monomer further imparts ionic conductivities comparable to liquid electrolytes on the order of 0.54 mS/cm (PVIm–1.0) and 1.26 mS/cm (PVIm–0.5).

**Figure 3:**
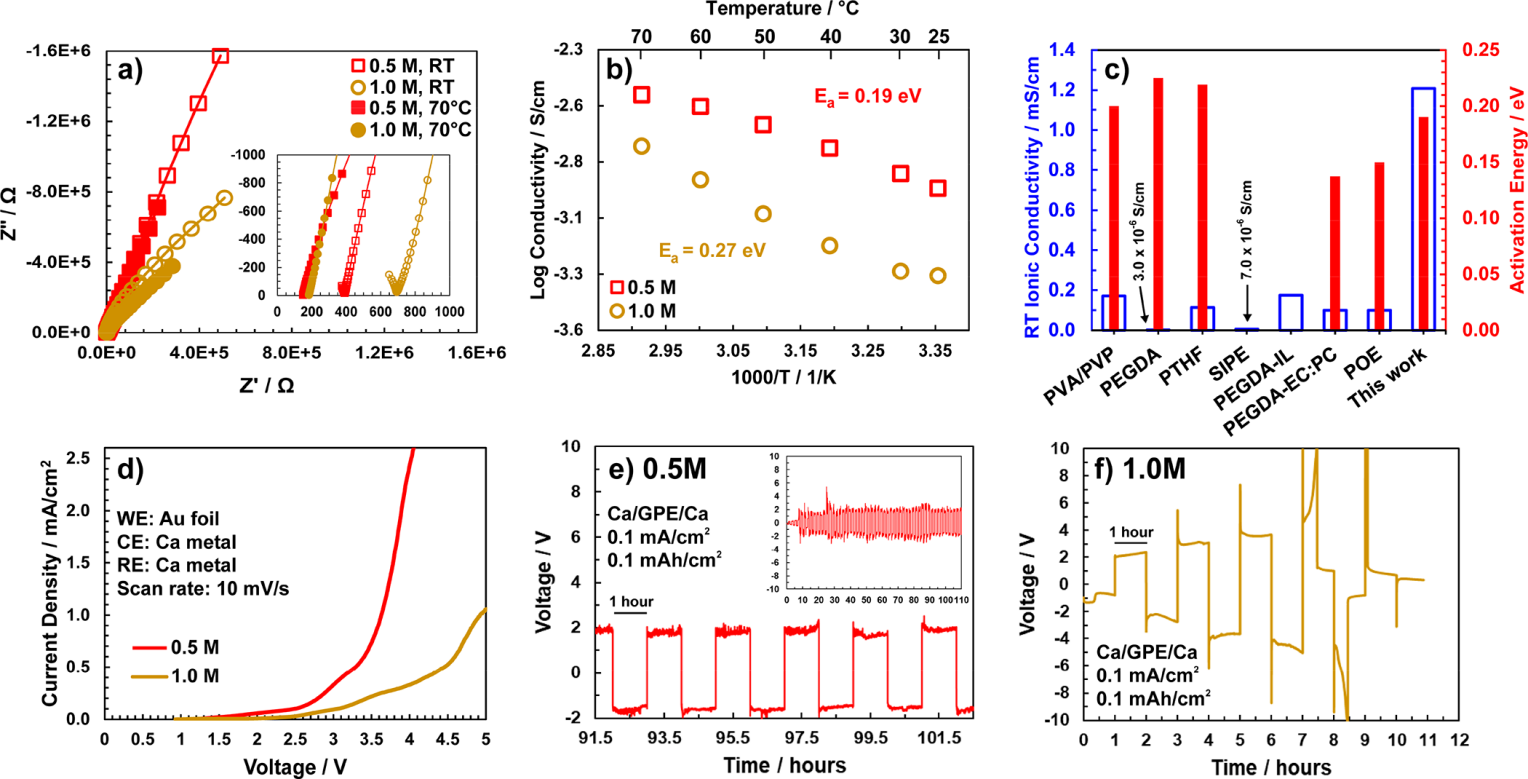
(a) Complex plot of the PVIm electrolytes containing 0.5 and 1.0 M Ca(TFSI)_2_ salt. Data at room temperature (ca. 25 °C) and 70 °C is shown (inset shows the high-frequency region of the complex plot). (b) Conductivity plot of the two salt concentrations with the indicated activation energies obtained via linear fits of the data. (c) Bar plot comparing room temperature (RT) ionic conductivities and activation energies for Ca–ion conduction. (d) Linear sweep voltammetry of the polymer electrolytes using Au foil as the working electrode. (e) Galvanostatic cycling of a Ca/Ca symmetric cell using PVIm–0.5 (inset shows the entire duration of cycling at the same current density). (f) Galvanostatic cycling of a Ca/Ca symmetric cell using PVIm–1.0. Data in (c) is obtained from refs (41, 8, 9, 13, 12, 14, and 10). Missing bars for activation energies in (c) indicate that data was not available.

Regarding moisture in the electrolyte, a small, broad peak at ∼3600 cm^–1^ was detected for PVIm–0.1, which belongs to the O–H vibration from moisture^33^ in the sample due to handling in the ambient. No other samples demonstrated this feature. The associated charge delocalization with the imidazole groups along the polymer backbone and charge delocalization on the salt anion are both conducive for softening cation–polymer interactions and decreasing the energetic barrier to ion hopping. Notably, the coordinating site in the monomer and that in the polymer is the same, i.e., the pyridine-type N atom. As such, the metal–ligand interactions herein can be explained on the basis of the hard and soft acid and base (HSAB) theory. Calcium, like lithium and magnesium, can be considered a hard electron acceptor^34^ (i.e., hard acid) and oxygen, a hard electron donor (i.e., hard base), the latter being as such in polymer electrolytes.^35^ Hard acids and hard bases are known to preferentially coordinate with one another. Thus, it is well established that the ether oxygen atom in PEO coordinates strongly with metal ions from the salt, thereby limiting long-range cation transport within the polymer.^36^ Nitrogen, however, is a somewhat milder form of a hard electron donor (i.e., milder hard base). The conjugated five-membered imidazole ring imparts polarizability to the nitrogen atom, lowering its basicity and making it a softer base than oxygen. Consequently, the strength of N–Ca^2+^ coordination becomes lower, and Ca^2+^ ion hopping is energetically more favorable. Furthermore, the tendency for hard acids such as calcium and magnesium to preferentially coordinate with oxygen over nitrogen in a variety of environments is also well-known,^37−40^ which is due to the ability of oxygen to coordinate with metal ions more strongly. The distance between hopping sites is also an important aspect in the context of ion motion. The distance between any two consecutive oxygen atoms in a PEO chain is 4.39 Å (calculated on the basis of the O–C–C–O bonds of the backbone), whereas the imidazole pendant groups in PVIm are separated by a distance of 3.06 Å (calculated on the basis the C–C–C bonds of the backbone). We note that, in previous computational studies on Li–PEO complexes, the O–O distance (2.9 Å) on a PEO chain has been taken as the hopping distance for Li^+^ ions. Our estimates for the intramolecular O–O and N–N bond distances (see Figures S8 and S9) yielded values of 2.65 and 1.44 Å, respectively; i.e., the hopping distance for calcium ions along a PVIm chain is nearly half that along a PEO chain. This closer spacing in PVIm possibly enables higher ionic conductivity. Hence, the combination of softer coordination and closely spaced coordination sites is reflected in the activation energy values calculated on the basis linear fits of the data. The activation energy for PVIm–0.5 is 0.19 eV, whereas that for PVIm–1.0 is 0.27 eV. Between the two concentrations explored, PVIm–1.0 exhibits lower ionic conductivity and a higher activation energy associated with hopping compared to that of PVIm–0.5. An increase in imidazole–Ca^2+^ coordination in the polymer plays a dual role: (1) it can result in higher ionic conductivity via a greater number of charge carriers, and (2) it increases the extent of polymer cross-linking., i.e., higher monomer conversion and more solid polymer content, which can reduce the ionic conductivity. Therefore, there exists a balance between the two factors (herein achieved with 0.5 M salt concentration, PVIm–0.5). Hence, the combination of salt concentration and polymer conversion are tunable parameters within this imidazole polymer chemistry, which can be investigated in the context of other salts and other imidazole-based monomers (e.g., via tuning the R group) to inform one of the optimum parameters for ionic conductivity and other electrolyte properties. Imidazole itself is a solid at room temperature with different R groups presenting it in a liquid state (herein, a vinyl group). Future work can explore the combination of polymerizable vinylimidazole with fractions of imidazole solvent with other R groups (e.g., methyl imidazole, ethyl imidazole) to further tune the electrolyte properties. Overall, a high ionic conductivity for both PVIm–0.5 and PVIm–1.0 demonstrates the superior conductivity of imidazole-based electrolytes for calcium ions and an advance over polymer electrolytes thus far reported in the literature.^8−10,12−14,41^

A comparison of conductivity and activation barrier values with current reports in the calcium polymer electrolyte literature is shown (Figure 3c). The highest values reported by these authors (and us) at RT are used for the purpose of comparison. Vanitha et al.^41^ investigated poly(vinylalcohol) (PVA)/poly(vinylpyrrolidone) (PVP)-based solid polymer electrolytes containing CaCl_2_ salt and demonstrated an RT ionic conductivity of 0.17 mS/cm. It is noteworthy that such ionic conductivity was achieved using polymers with *T*_g_ above 100 °C. Our group then investigated poly(ethylene glycol diacrylate) (PEGDA)-based^8^ and poly(tetrahydrofuran) (PTHF)-based^9^ solid polymer electrolytes containing various salts, which demonstrated RT ionic conductivities of 3.0 × 10^–6^ and 1 × 10^–4^ S/cm, respectively. Followed by this, Ford and co-workers^13^ were the first to report a gel-type single-ion conducting polymer for calcium ion conduction. We note that, since the anions are covalently tethered to the polymer chains in single-ion conducting polymers, they do not partake in ion transport. Therefore, ion conductivity in single-ion conducting polymers is primarily from the cation. Using a poly(tetrahydrofuran)–diacrylate polymer matrix (PTHFDA), these authors reported a remarkable RT ionic conductivity of 7 × 10^–6^ S/cm. Subsequent efforts, mostly by our group, were centered around developing gel–polymer electrolytes incorporating ionic liquids^12^ and cyclic carbonate solvents^14^ into ether-based polymer matrices, with both strategies proving largely successful. Notably, the liquid content in PVIm–0.5 and PVIm–1.0 herein is comparable to that previously reported in other systems. In our previous reports, EC/PC^14^ and ionic liquid^12^ solvent content in PEGDA matrices was 50 wt % and the ionic conductivity was ∼10^–4^ S/cm. On the other hand, Ford et al.^13^ in their SIPE system reported a swelling-induced volume change of the polymer electrolyte between 30.6% and 164.5% depending on the solvent used. More recently, Martinez-Cisneros et al.^10^ reported an RT ionic conductivity of 0.1 mS/cm in a solid polymer electrolyte using a polyoxyethylene backbone. Schauser et al.^18,22^ investigated the ionic conductivity of imidazole-based polymer electrolytes containing M–TFSI_*x*_ (M = Cu^2+^, Ni^2+^, Zn^2+^, and Fe^3+^) salts and did not find improvement in total ionic conductivity relative to the PEO-based ones. Departing from the trend of using O-containing polymers, the present work is the first to employ an imidazole-based polymer for calcium ion conduction, wherein the synergistic effect of soft cation–polymer interactions and the ability of Ca^2+^ to bridge pendant imidazole groups, which themselves serve as soft coordinating sites, facilitates high ionic conductivity and a suitable energy of activation for ion hopping. Importantly, the activation energy for ion hopping in PVIm electrolytes shown herein is lower than several incumbent ether-based candidates. Collectively, these results outline the promising nature of imidazole-based electrolytes for enhanced calcium ion conduction compared to their ether-based counterparts.

Finally, we assess the performance of the polyvinylimidazole (PVIm) electrolytes to reveal their potential use in electrochemical cells. The oxidative stability of the polymer electrolytes was found to be ∼2.7 V for PVIm–0.5 and ∼3.2 V for PVIm–1.0 (Figure 3d). Complete salt dissolution and a higher degree of cross-linking in the polymer leads to an increase in the oxidative stability of the electrolyte.^12,14^ This increased conversion and cross-linking, indicated by thermal analysis, imparts slightly better oxidative stability to PVIm–1.0. For reference, in our previous work using an imidazolium-based ionic liquid trapped within a PEGDA matrix,^14^ we observed a similar oxidative stability of ∼3 V. We note that the use of calcium metal as the reference and counter electrode can shift the potential and current density observed during voltammetry measurements. Yet, calcium metal was used in this work owing to the standard practice of doing so in the literature and the difficulty of placing a separate reference electrode given the size of the polymer samples investigated. A representative cathodic scan of the PVIm–0.5 electrolyte revealed that the electrolyte is reductively stable up to at least −2 V (see Figure S10). The electrochemical cycling performance of the polymer electrolytes in galvanostatic mode was determined in symmetric Ca//Ca cells. We chose a nominal current density of 0.1 mA/cm^2^ in this work, which is common in the calcium battery electrolyte literature. PVIm–0.5 cycled for over 100 h at an areal capacity of 0.1 mAh/cm^2^ and at overpotentials of −1.8 and +2 V (see Figure 3e and its inset), which is attributed to the high ionic conductivity of the electrolyte. Furthermore, this also signifies the formation and presence of a stable solid electrolyte interface (SEI) layer on the calcium metal working electrode surface, which facilitates stable galvanostatic deposition and dissolution of calcium metal. It appears that the SEI forms in the initial 3 cycles^6,11,14^ wherein the overpotentials are ca. −0.5 and +0.5 V, and the overpotentials stabilize at higher values. On the other hand, PVIm–1.0 demonstrated only 3 full cycles (see Figure 3f) before reaching very high overpotentials. Further, sharp spikes are observed upon switching the polarity cycle 2 onward, which is likely due to concentration polarization effects within the polymer electrolyte.^42^ For reference, cycling overpotentials reported with recent Ca^2+^-conducting liquid electrolyte systems are in the range of 0.1 to 1.0 V with lower overpotentials attributable to the Ca[B(hfip)]_2_ salt. The reader is referred to recent review articles for detailed information on the state-of-the-art.^43,44^ An unstable interface at the working electrode surface and effects arising from the complex ion-pair formation (aggregates) at higher salt concentrations can also contribute to high overpotentials. Noise in the plateaus during galvanostatic cycling may also originate from localized current densities due to irregularities on the surface of the working electrode. Indeed, the surface of PVIm–1.0 was found to be rougher compared to that of PVIm–0.5 on the basis of SEM analysis of the surfaces of the polymer electrolytes (see Figure S11). The stability of the monomer and VIm–0.5 resin against a Ca metal surface at 0 V conditions was also investigated. Flat, polished Ca metal pieces were fully submerged in 1-vinylimidazole monomer and VIm–0.5 resin for 24 h in an inert environment (<0.5 ppm of H_2_O and O_2_). Surface FTIR of the recovered Ca metal pieces exhibited traces of the pure monomer or the resin but no distinct decomposition products (see Figure S12). This suggests that the monomer and electrolyte are stable against Ca metal when at 0 V. Since the decomposition of Ca(TFSI)_2_ salt has been established in the literature, we posit that the high overpotentials are a consequence of sluggish electrode kinetics as well as the products formed due to salt decomposition. Although Ca(TFSI)_2_ is generally less prone to ion pair formation due to better charge delocalization on the TFSI^−^ anion, it can still form ion pairs in solution at high salt concentrations,^5^ and calcium deposition and dissolution depend heavily upon the coordination environment of Ca^2+^ in the electrolyte.^45,46^ For example, the existence of contact ion pairs in the electrolyte lowers the cation transference number and increases the energy required for desolvation,^47^ which necessitates a higher overpotential for metal deposition. Lastly, the increasing overpotentials seen with PVIm–1.0 are likely an effect of increasing SEI and/or charge-transfer impedances, which we have shown previously via systematic impedance spectroscopy-based studies during galvanostatic steps.^6,11,14^ Suitable approaches to tuning cation–polymer interactions and improving the interfacial electrochemistry, including the study of other salts, are currently being explored and will be reported in the future.

In summary, we have synthesized and characterized an imidazole-based polymer electrolyte for calcium ion conduction using a photopolymerization approach, which to the best of our knowledge has so far not been reported in the literature. These electrolytes demonstrate impressive room temperature ionic conductivities of 0.54 and ∼1 mS/cm based on salt concentration, attributable to soft N–Ca^2+^ coordination based on the polarizability of the imidazole pendant groups, which reduces the Lewis basicity of the coordinating N atoms. Ion hopping appears to be the primary mechanism of ion conduction in these polymer electrolytes owing to their glassy, amorphous structure at room temperature, and we further postulate that long-range ion transport may be facilitated by the synergistic combination of labile interchain cross-links formed by calcium ions and the presence of low viscosity liquid vinylimidazole in the polymer matrix. Furthermore, preliminary electrochemical cycling using these polymer electrolytes demonstrated promising overpotentials at reasonable capacities. This work establishes the use of a non-ether, imidazole-based coordination strategy for calcium polymer electrolytes and lays the foundation for the development of imidazole-based electrolytes for calcium ion conduction.

## Experimental Methods

### Materials

1-Vinylimidazole monomer (99%) and calcium metal (98%, granular) were purchased from Sigma–Aldrich, USA, and used as received. Gold (Au) foil was purchased from Goodfellow, Inc., and used as received. Ca(TFSI)_2_ salt was purchased from Alfa–Aesar, USA, and dried under vacuum for 16 h at 117 °C before use. Irgacure 784 photoinitiator was purchased from BASF Chemicals, Germany, and used as received. The monomer and salt were stored in an argon-filled glovebox with moisture and oxygen levels of <0.5 ppm. The photoinitiator was stored under ambient conditions and shielded from light.

### Photopolymerization

**Scheme 1:**
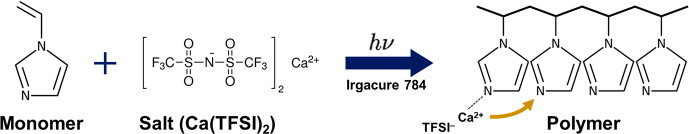
Preparation of the Poly(vinylimidazole) Electrolytes via Photopolymerization and Proposed Ion Transport Mechanism

As shown in Scheme 1, appropriate amounts of dry salt were dissolved in the monomer along with 1.0 wt % photoinitiator to obtain photopolymerizable mixtures. All weighing steps (except the photoinitiator), mixing steps, and photopolymerization steps were carried out inside an argon-filled glovebox. The mixtures were protected from ambient light and stirred continuously at a temperature of ∼50 °C to ensure thorough mixing of the salt in the monomer. Before photopolymerization, the mixtures were allowed to cool to room temperature. Photopolymerization of the room temperature mixtures was then carried out using a high-power UV LED lamp (Thorlabs, Inc.) operating at a wavelength of 365 nm and irradiation intensity of ∼125 mW/cm^2^. The samples were fully cured after 4 h of exposure time. Specifically, 0.25 mL of the liquid mixture was contained in home-made PTFE rings (1 mm thick, 17 mm in diameter) glued to a Corning borosilicate glass substrate. The contained mixtures were irradiated with the UV light from the bottom. The light intensity used herein was 20 times lower than that used in the literature, which necessitated the use of a longer exposure time.^25^ Photocuring inside the glovebox prevented the use of a higher light intensity lamp in this work. All samples were stored under ambient conditions after photopolymerization.

### Spectroscopy

Fourier transform infrared (FTIR) spectroscopy was carried out in ATR mode using a Nicolet iS5 spectrometer at a resolution of 4 cm^–1^ with 16 scans per collection. For liquid mixtures, spectra were obtained by directly placing ∼40 μL of the mixtures onto the diamond window of the instrument. Background subtraction, normalization, and peak deconvolution of the spectra were performed using the OriginLab Pro software package.

### Polymer Characterization

Thermogravimetric analysis (TGA) of the polymer samples was carried out in platinum pans using a TA Instruments Q500 analyzer under N_2_ flow from room temperature to 600 °C at a ramp rate of 10 °C/min. Differential scanning calorimetry (DSC) was carried out using a TA Instruments Q200 calorimeter between −50 and 300 °C. For both TGA and DSC measurements, sample weights were between 1.5 and 8 mg. X-ray diffraction (XRD) of the polymer samples was carried out using a Rigaku MiniFlex diffractometer and Cu Kα radiation. Scanning electron microscopy (SEM) was carried out on a JEOL JSM-IT100LA instrument at an accelerating voltage of 5 kV. Gel permeation chromatography (GPC) of the polymer samples was carried out using an Agilent Technologies 1260 instrument equipped with triple detectors (refractive index, viscosity, and light scattering). Millipore water containing 0.1 M NaNO_3_ and 2% (v/v) acetic acid was used as the eluent at a flow rate of 0.7 mL/min. Three columns were used: Supelco G5000PW_XL_, 4000, and 2000 with their temperatures maintained at 30 °C. The d*n*/d*c* value used for the calculation of molecular weights was 0.22 mL/g as reported in the literature.^48^

### Electrochemical Characterization

Electrochemical impedance spectroscopy (EIS) was carried out using a Solartron EnergyLabXM instrument with stainless steel blocking electrodes at an amplitude of 10 mV and in the frequency range of 1 MHz to 0.1 Hz. Spectra were first collected at room temperature and then between 30 and 70 °C in steps of 10 °C. An 18-minute soak was applied to the test cells at each temperature to ensure thermal equilibration of the polymer samples. Using the same instrument, linear sweep voltammetry (LSV) was carried out in a 2-electrode setup using gold foil as the working electrode and flat, polished calcium metal as both the counter electrode and the reference electrode at a scan rate of 10 mV/s. Cationic transference number measurements were performed using the method described previously.^9^ Lastly, 2-electrode galvanostatic cycling on symmetric Ca/Ca cells was performed on a VersaSTAT 3 or a Solartron EnergyLabXM instrument using flat, polished calcium metal pieces as the electrodes with the polymer samples sandwiched between them. Flat calcium metal pieces were obtained by pressing irregular granular pieces of calcium metal between flat stainless steel plates under a hydraulic press, after which they were transferred into the glovebox. Polishing of these flat calcium pieces was carried out using a Dremel rotary tool fitted with a fine stainless steel wire brush until a reflective mirror-finish was obtained. The applied current density for all samples was ∼0.1 mA/cm^2^. For all electrochemical studies, small rectangular samples of the polymers measuring 2 mm (*W*) × 3 mm (*L*) on average were used. The dimensions were measured after the EIS measurement to account for any changes in the polymer owing to its softness. Thicknesses of polymer samples were in the range of 200–350 μm.

## Supporting Information Available

Figure S1: full FTIR spectra for the neat monomer, pure salt, liquid mixtures before polymerization, and electrolytes obtained after polymerization; Figure S2: magnified FTIR data; Figure S3: FTIR data for the O=S=O vibration of the salt; Figure S4: TGA curves of neat monomer and salt-containing polymers; Figure S5: DSC exotherms of neat monomer and salt-containing polymers; Figure S6: XRD profiles of neat monomer and salt-containing polymers; Figure S7: room temperature Nyquist plot of a polymer without salt (PVIm–0.0); Figure S8: intramolecular O–O bond distance for a PEO chain; Figure S9: intramolecular N–N bond distance for a PVIm chain; Figure S10: representative cathodic scan of a PVIm–0.5 polymer electrolyte; Figure S11: SEM micrographs of polymers; Figure S12: FTIR data for Ca metal pieces submerged in neat monomer and VIm–0.5 resin; Figure S13: representative TGA curves of PVIm–0.5 samples with varying liquid content (PDF)
