# Supplementary material for: Vinylimidazole-Based Polymer Electrolytes with Superior Conductivity and Promising Electrochemical Performance for Calcium Batteries
Source: ACS Appl Polym Mater. 2022 Sep 12;4(10):6803–11. doi: 10.1021/acsapm.2c01140 (PMC9578112; doi:10.1021/acsapm.2c01140)
Supplement: Supplementary file 1 — ap2c01140_si_001.pdf [file ap2c01140_si_001.pdf]

Supporting Information

Vinylimidazole–based Polymer Electrolytes with Superior Conduction and Promising Electrochemical Performance for Calcium Batteries

Shreyas Pathreeker[1] and Ian D. Hosein*[1]

[1]Department of Biomedical and Chemical Engineering, Syracuse University, Syracuse, NY 13244

*Corresponding author: idhosein@syr.edu

1. **Fourier Transform Infrared Spectroscopy (FTIR)**

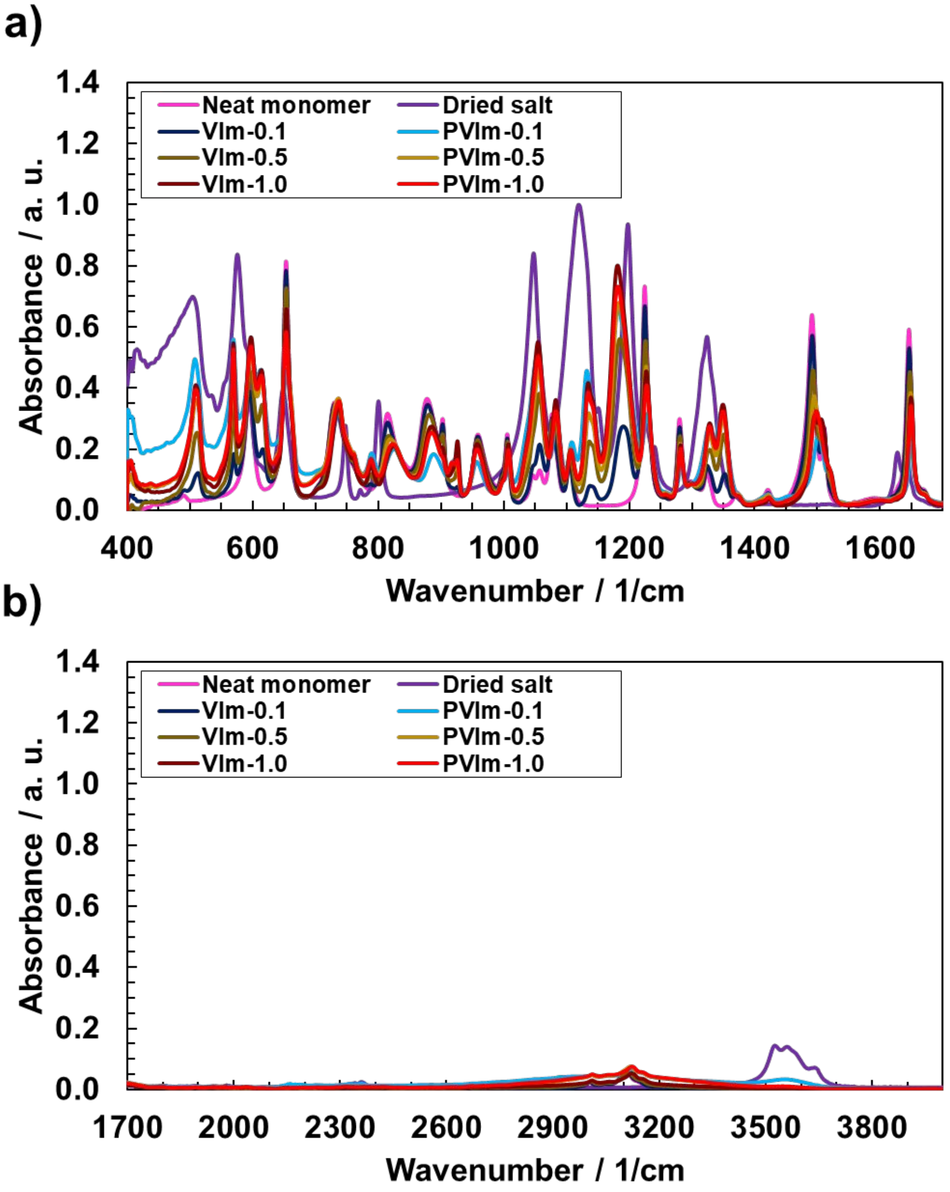

Figure S1. Full–spectrum FTIR data for all samples.

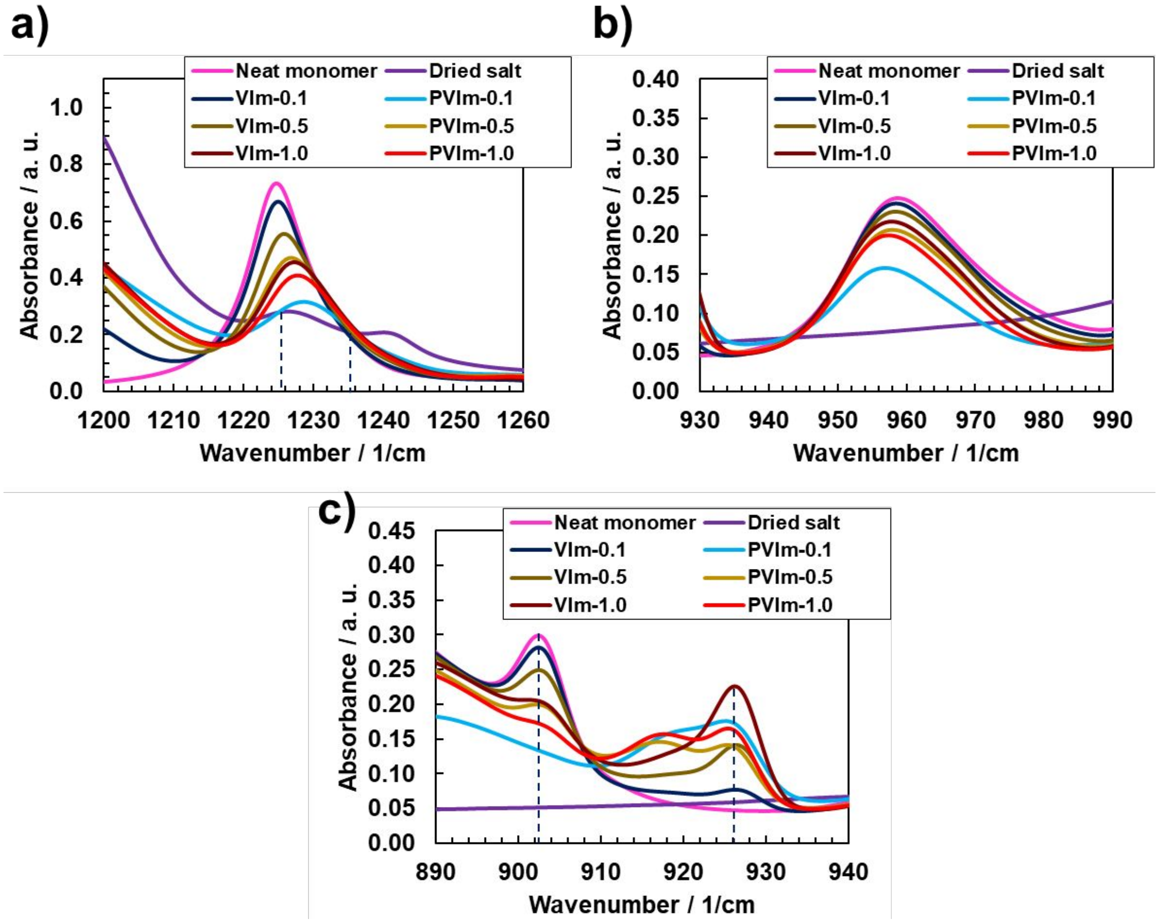

Figure S2. Magnified FTIR data for a) the 1200 cm^{-1} to 1260 cm^{-1} region, b) the 930 cm^{-1} to 990 cm^{-1} region, and c) the 890 cm^{-1} to 940 cm^{-1} region.

Notably, the vibrational mode associated with the salt S–N–S bond found at 740 cm^{-1} is more sensitive to changes in coordination environment, and is therefore considered more suitable for coordination analysis. However, we were unable to use this peak for coordination analysis owing to its overlap with the C–N–C bond signature of the imidazole ring.

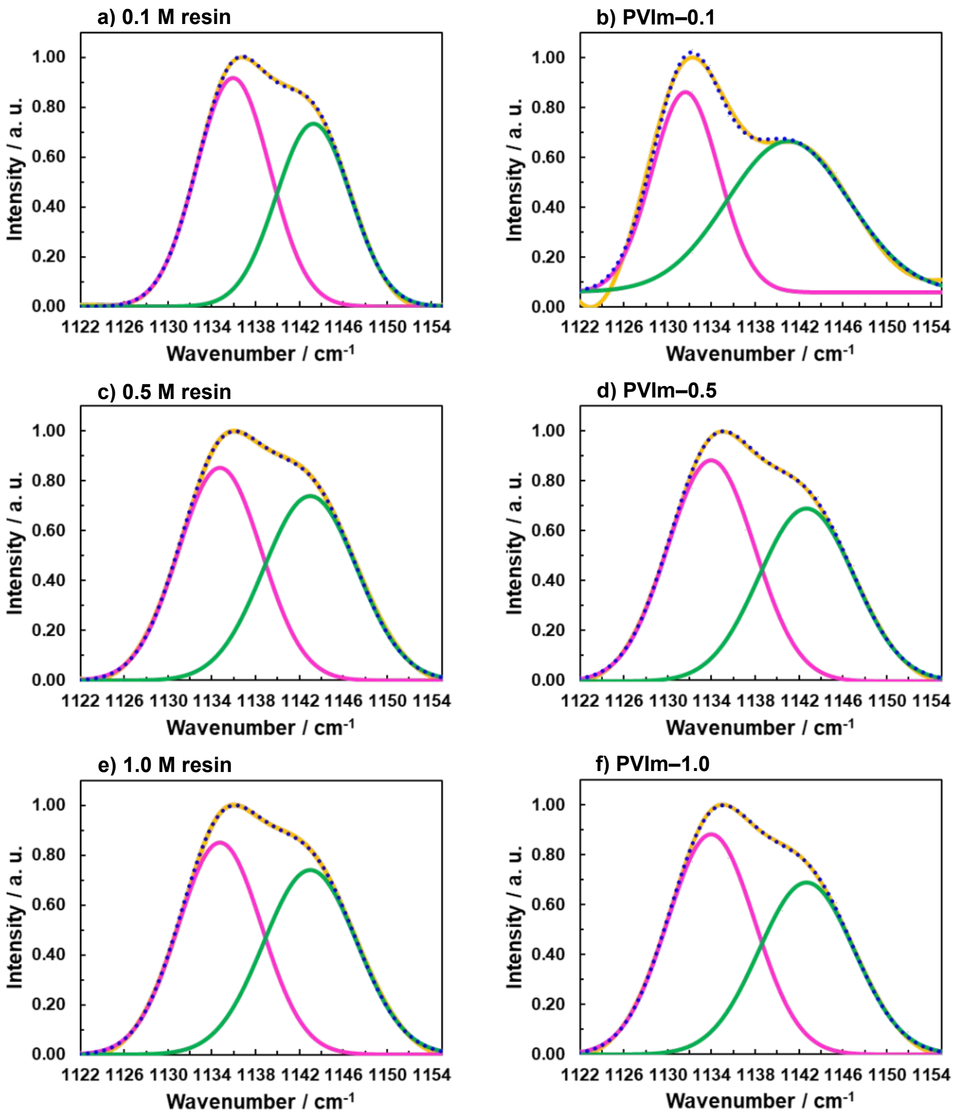

Figure S3: FTIR data for the SO_2 vibration peak associate with the salt for the three salt concentrations explored. First column shows data for the resins, and second columns shows data for polymers. Peaks at ~1136 cm^{-1} represent unpaired TFSI$^-$ anions, whereas shoulders at ~1143 cm^{-1} indicate paired TFSI$^-$ anions.

2. Thermogravimetric Analysis (TGA), Differential Scanning Calorimetry (DSC), and X–ray Diffraction (XRD)

While all samples demonstrated imidazole–Ca^{2+} coordination and underwent photopolymerization, polymer films suitable for electrochemical analysis were obtainable only at salt concentrations of 0.5 M and 1.0 M. Therefore, all additional studies were focused on polymer electrolytes obtained using these two salt concentrations.

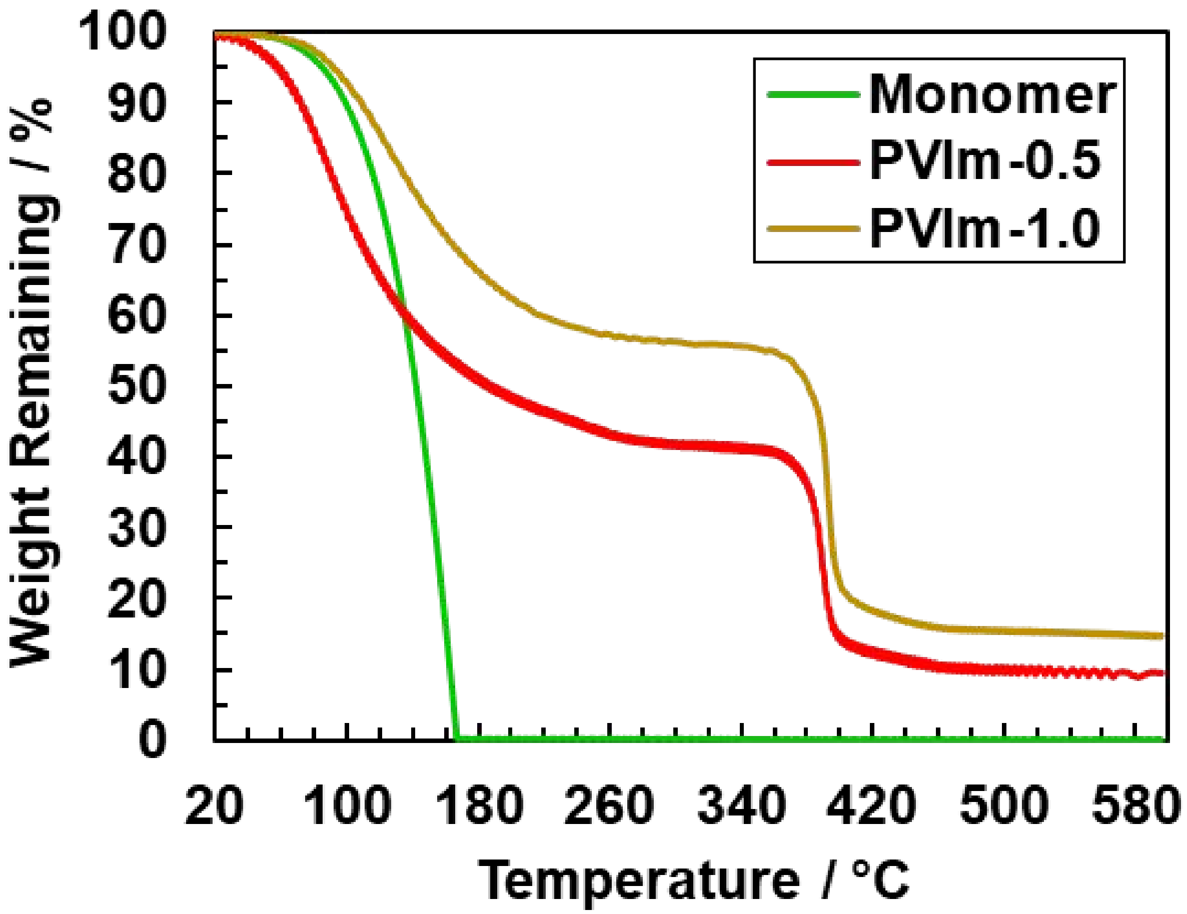

Figure S4: TGA curves for the neat monomer, PVIm–0.5, and PVIm–1.0 polymer electrolytes.

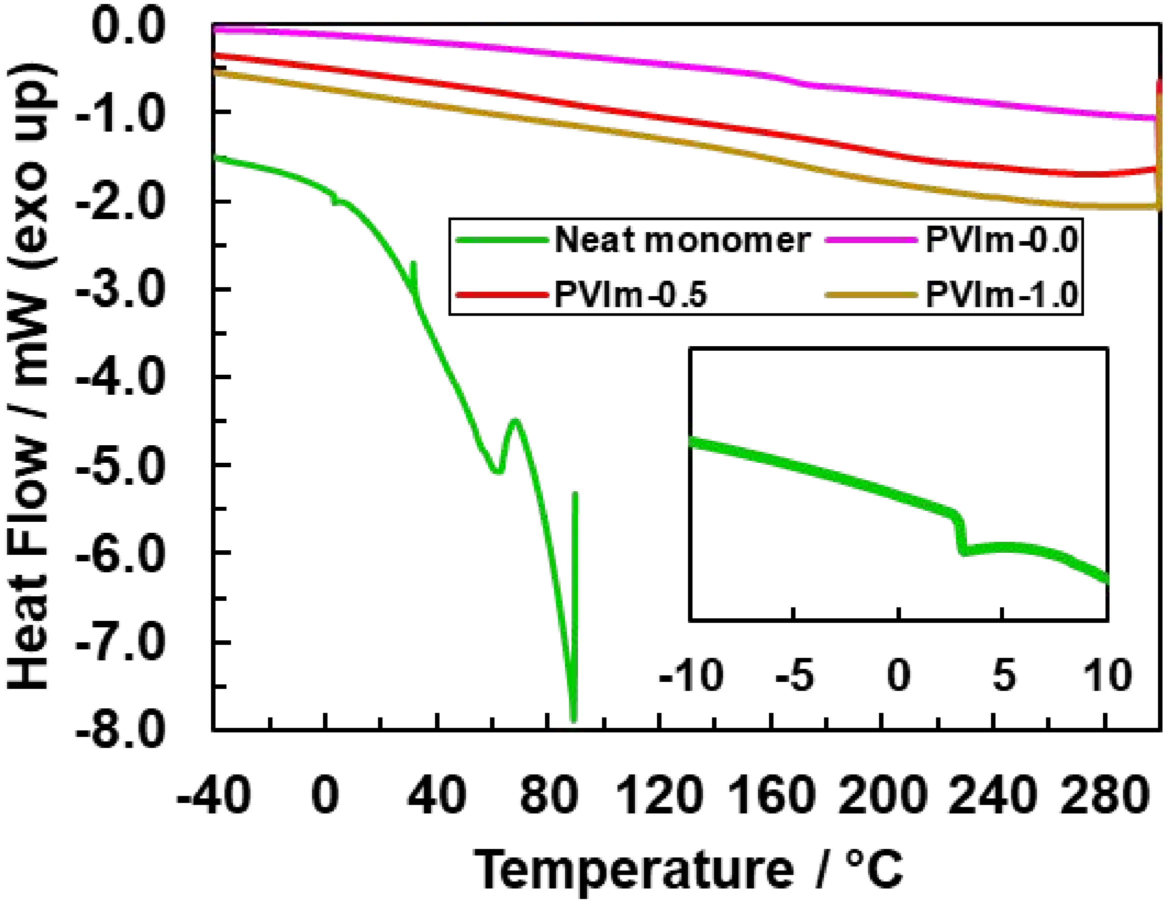

Figure S5: DSC data for neat monomer, PVIm–0.0, PVIm–0.5, and PVIm–1.0 polymer electrolytes. The first heating is shown for the monomer, whereas second heating curves are shown for the remaining samples.

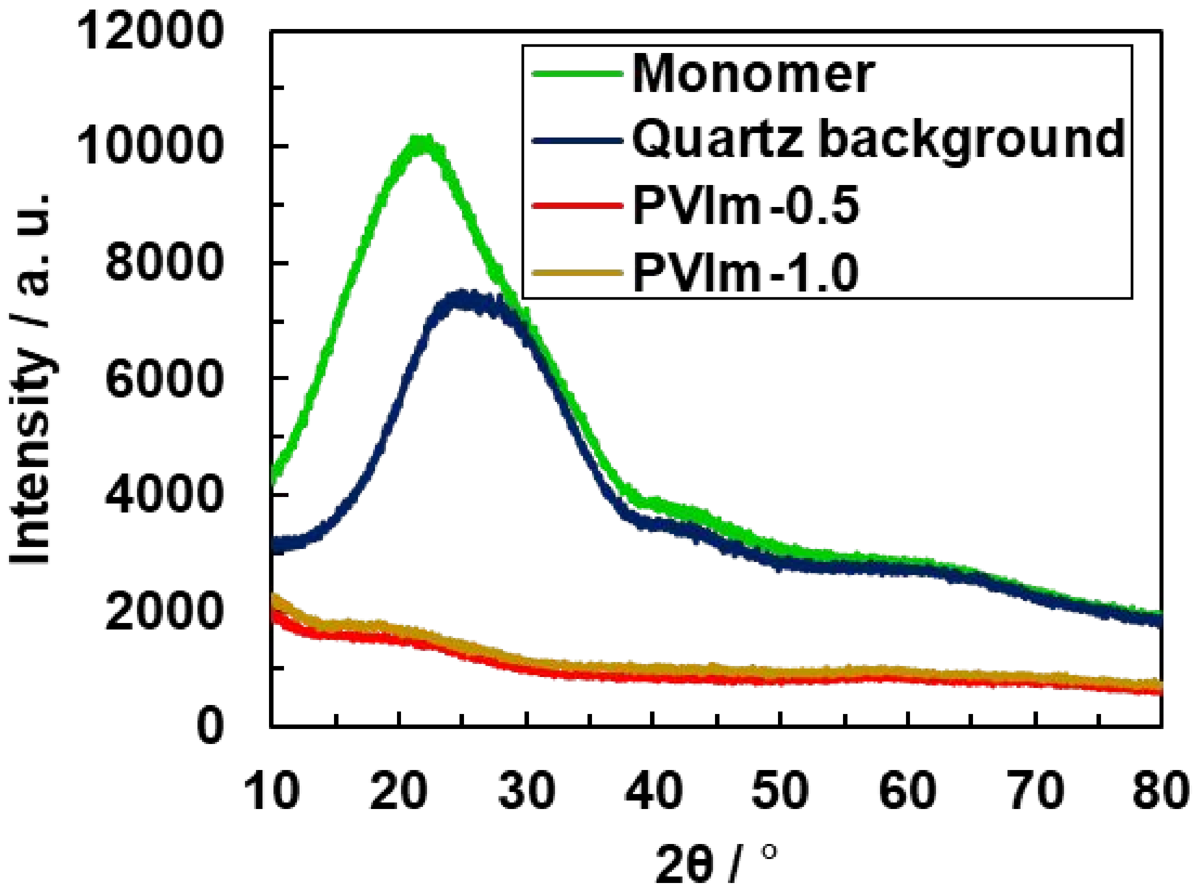

Figure S6: XRD profiles for the neat monomer, the quartz sample holder, PVIm–0.5, and PVIm–1.0 polymer electrolytes.

3. Electrochemistry

Intercepts of the high–frequency semi–circle with the x–axis are used to extract the bulk impedances associated with the polymer electrolytes. The electrochemical analysis was limited to a temperature of 70°C based on the thermal properties of the polymer revealed by TGA analysis. Additional room–temperature (RT) EIS measurements were performed using fresh PVIm–0.5 samples, which yielded similar or higher ionic conductivities. RT ionic conductivity of the liquid resin prior to polymerization was found to be 1.59 mS/cm. RT ionic conductivity of a PVIm – 0.0 (i.e., no salt) sample was found to be 1.7 x 10^{-5} S/cm (see below).

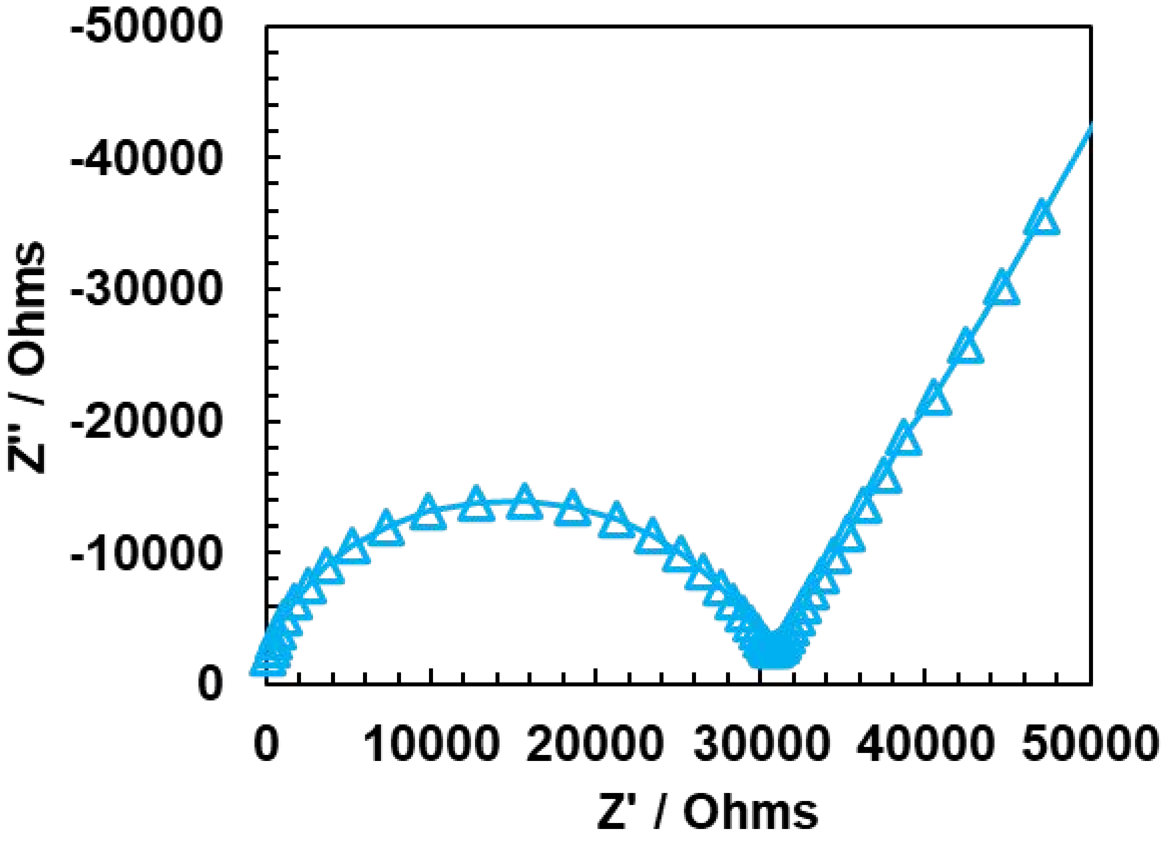

Figure S7: Nyquist plot of a PVIm–0.0 sample.

4. Intramolecular bond distances

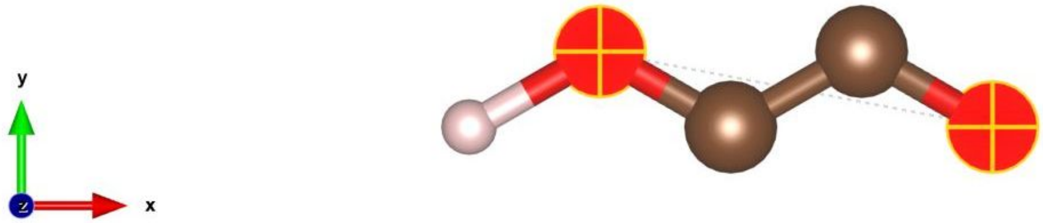


```
                     x          y          z          Occ.      U    Site      Sym.
   1 H   H1      15.32550  -10.26510    0.00000    1.000    0.000    1          -
   2 O   O1      16.19150   -9.76510    0.00000    1.000    0.000    1          -
   3 C   C1      17.05750  -10.26510    0.00000    1.000    0.000    1          -
   4 C   C2      17.92350   -9.76510    0.00000    1.000    0.000    1          -
   5 O   O2      18.78960  -10.26510    0.00000    1.000    0.000    1          -
==================================================================================

Number of polygons and unique vertices on isosurface = 0 (0)
5 atoms, 4 bonds, 0 polyhedra; CPU time = 0 ms

Atom:  2    O1   O  16.19150  -9.76510   0.00000  ( 0, 0, 0)+ x, y, z
          (x,y,z):  16.19150  -9.76510   0.00000
              Occ. = 1.000         Ueq = 0.00000      1       -
Atom:  5    O2   O  18.78960 -10.26510   0.00000  ( 0, 0, 0)+ x, y, z
          (x,y,z):  18.78960 -10.26510   0.00000
              Occ. = 1.000         Ueq = 0.00000      1       -

l(O1-O2) =  2.64578(0) Å
   2      O1  O 16.19150 -9.76510  0.00000 ( 0, 0, 0)+ x, y, z
   5      O2  O 18.78960 -10.26510  0.00000 ( 0, 0, 0)+ x, y, z
```

Figure S8: O–O bond distance estimation for a PEO chain (2.64 Å). Yellow crosshairs indicate the oxygen atoms between which the distance is estimated.

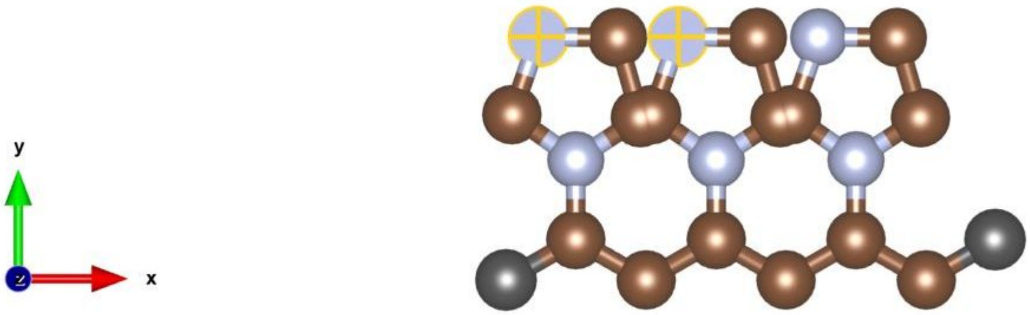


```
Atom:  2    O1   O  16.19150  -9.76510   0.00000  ( 0, 0, 0)+ x, y, z
          (x,y,z):  16.19150  -9.76510   0.00000
              Occ. = 1.000          Ueq = 0.00000      1        -
Atom:  5    O2   O  18.78960 -10.26510   0.00000  ( 0, 0, 0)+ x, y, z
          (x,y,z):  18.78960 -10.26510   0.00000
              Occ. = 1.000          Ueq = 0.00000      1        -

l(O1-O2) =  2.64578(0) Å
   2       O1  O 16.19150 -9.76510  0.00000 ( 0, 0, 0)+ x, y, z
   5       O2  O 18.78960 -10.26510  0.00000 ( 0, 0, 0)+ x, y, z

Atom: 23    N6   N  -2.19310   1.25620   0.00000  ( 0, 0, 0)+ x, y, z
          (x,y,z):  -2.19310   1.25620   0.00000
              Occ. = 1.000          Ueq = 0.00000      1        -
Atom: 10    N2   N  -0.74620   1.25620   0.00000  ( 0, 0, 0)+ x, y, z
          (x,y,z):  -0.74620   1.25620   0.00000
              Occ. = 1.000          Ueq = 0.00000      1        -

l(N6-N2) =  1.44690(0) Å
  23       N6  N -2.19310  1.25620  0.00000 ( 0, 0, 0)+ x, y, z
  10       N2  N -0.74620  1.25620  0.00000 ( 0, 0, 0)+ x, y, z
```

Figure S9: N–N bond distance estimation for a PVIm chain (1.44 Å). Yellow crosshairs indicate the nitrogen atoms between which the distance is estimated.

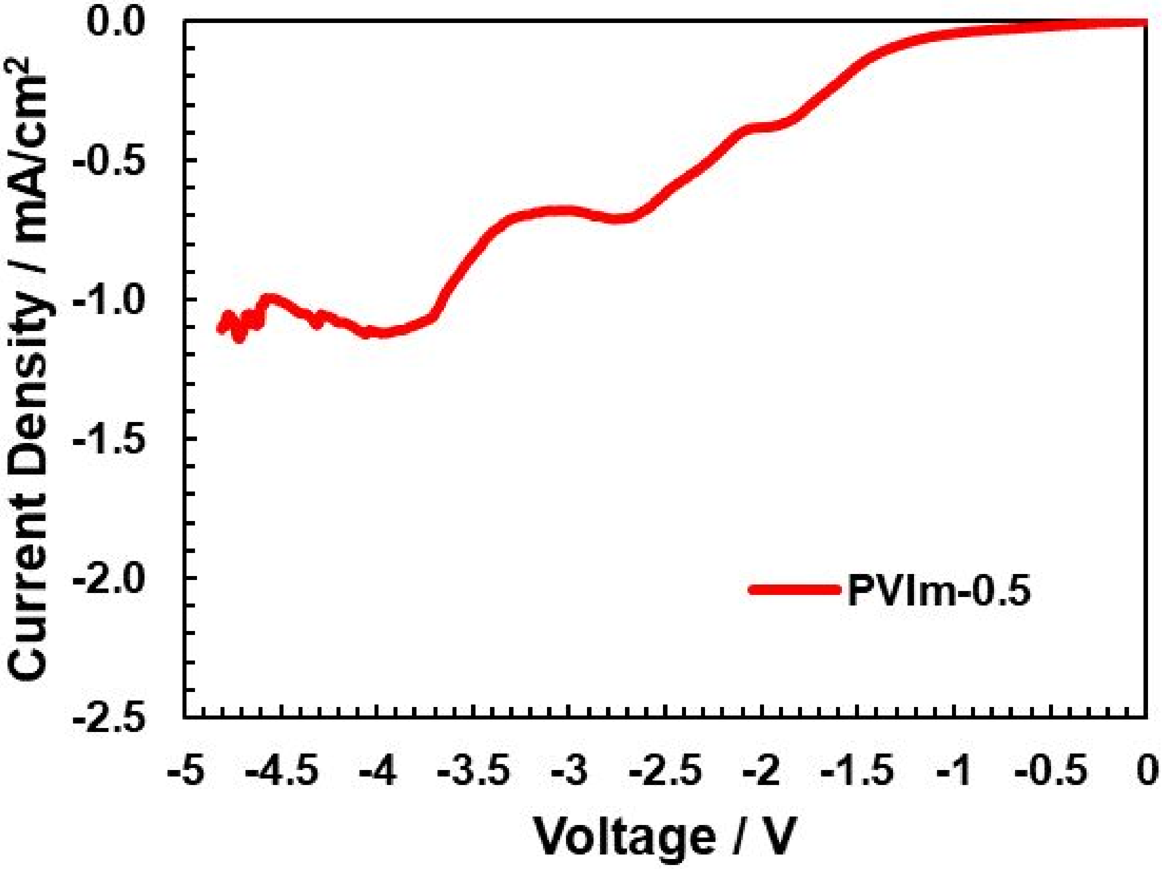

Figure S10. Representative cathodic sweep of a PVIm–0.5 polymer electrolyte showing its reductive stability.

5. Scanning Electron Microscopy (SEM)

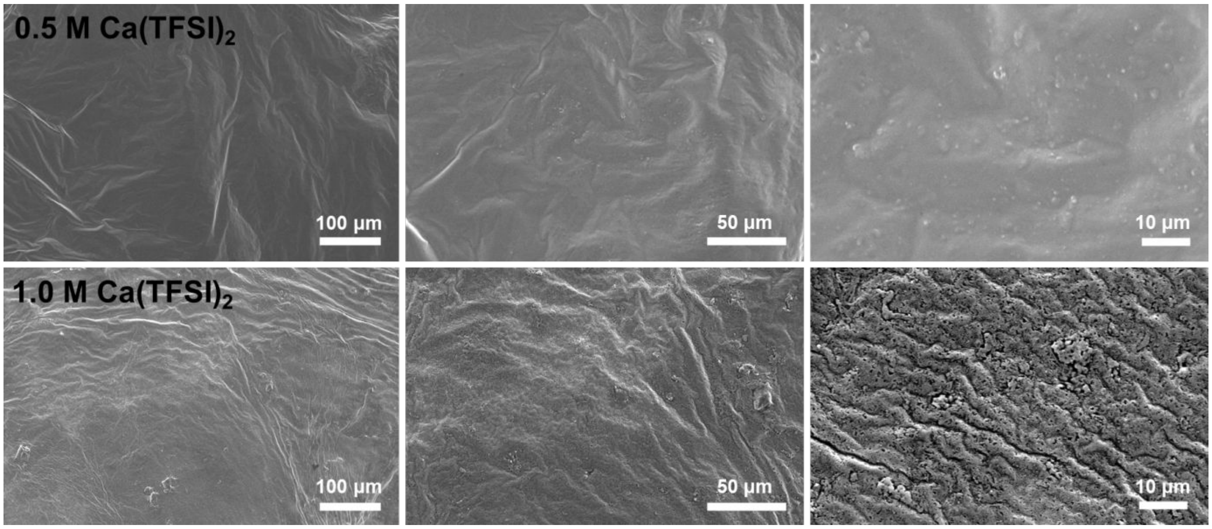

Figure S11: Top–down SEM images of the surfaces of the PVIm–0.5 and PVIm–1.0 polymer electrolytes.

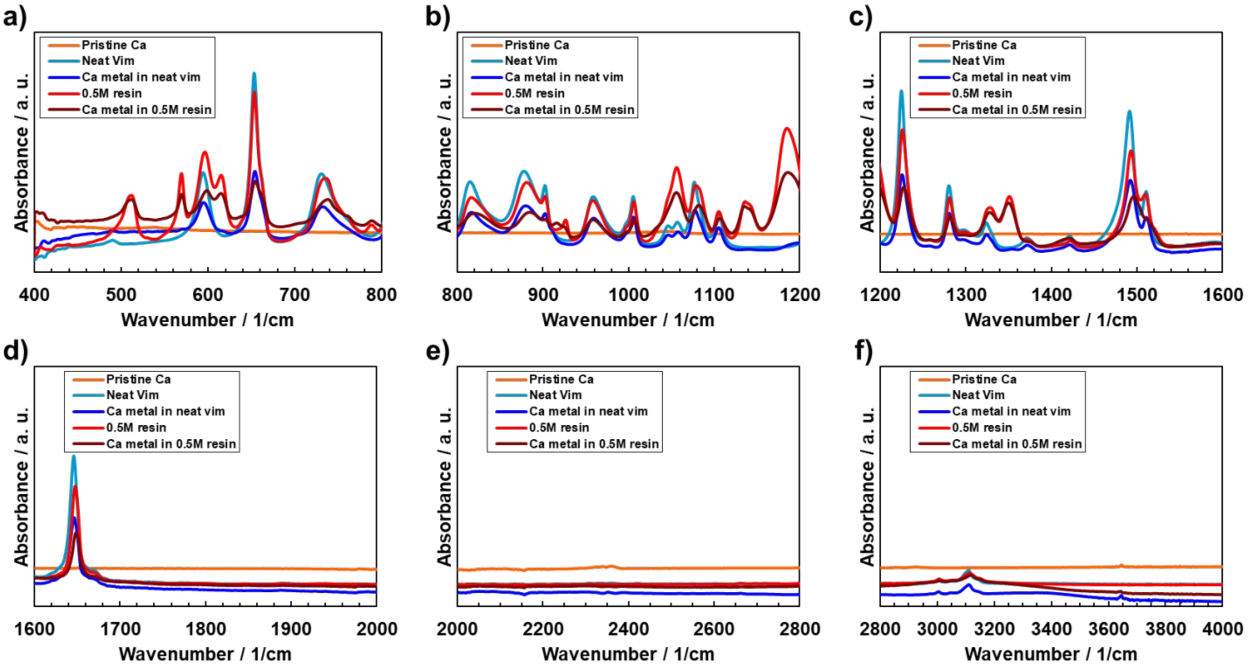

Figure S12: Surface FTIR data for polished Ca metal pieces recovered from neat monomer and Vim–0.5 resin following a 24 hour submergence experiment.

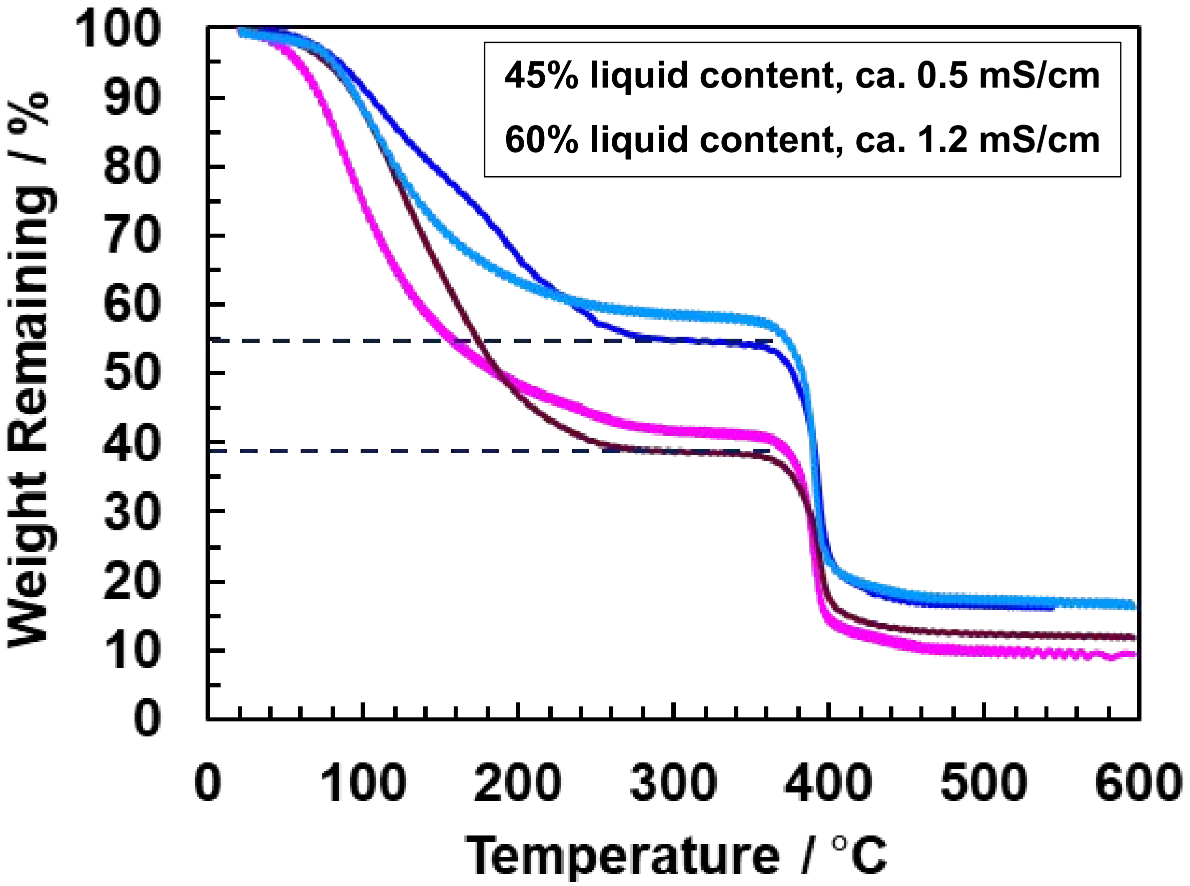

Figure S13: Representative TGA profiles for PVIm–0.5 polymer electrolytes synthesized with varying liquid content to outline the correlation between RT ionic conductivity and liquid content.
